# Clinical outcomes of metallic Y-shaped covered stents for bronchopleural fistula around upper carina after lobectomy

**DOI:** 10.1186/s12890-019-0973-9

**Published:** 2019-11-05

**Authors:** Yonghua Bi, Xiaoyan Zhu, Zepeng Yu, Gang Wu, Xinwei Han, Jianzhuang Ren

**Affiliations:** 1 Department of Interventional Radiology, the First Affiliated Hospital of Zhengzhou University, No.1, East Jian She Road, Zhengzhou, 450052 China; 2 Department of Histology and Embryology, College of Basic Medicine, Zhengzhou University, Zhengzhou, China

**Keywords:** Respiratory tract fistula, Stents, Fluoroscopy, Postoperative complications

## Abstract

**Background:**

Few studies have reported the placement of metallic Y-shaped covered stents (Y stents) for bronchopleural fistula around the upper carina.

**Methods:**

Eighteen patients were treated with Y stents insertion under the guidance of fluoroscopy. All covered stents were custom-designed and inserted to fit the upper carina anatomy. Clinical data and medical imaging data were analyzed retrospectively.

**Results:**

The stents were implanted successfully for the first time in 17 patients, and one patient needed a second attempt due to stent migration during withdrawal of the guide wires. In total, 19 small Y single-plugged stents were inserted in the upper carina and 5 large Y stents additionally in the main carina. Nineteen complications were observed in 14 patients, including 4 major complications. Stents were successfully removed in 12 patients due to complications or cure efficacy, for a median duration in place of 89.5 days. One patient lost follow-up. Nine patients were cured, and three had clinical improvement. One patient died of ventricular fibrillation the second day after the procedure and 4 patients died of tumors 7.8 to 91.7 months after stent placement. The 1-, 3-, and 5-year survival rates were 87.5, 80.8 and 80.8%, respectively.

**Conclusions:**

Metallic Y stent placement is technically feasible, effective and safe for bronchopleural fistula disease around the upper carina.

## Background

Metallic airway stent placement has proven to be an effective treatment for patients suffering from airway fistula or airway stenosis [1–4], since its first application reported by Simonds et al. in 1989 [5]. Use of metallic Y-shaped covered stents (Y stents) in bronchopleural fistula around the main carina has been reported [6]. Oki et al. used silicone stents to treat airway disease around the primary right carina [7]. However, they later reported that metallic Y stents inserted with guide wire support were less traumatic, which is an alternative for lesions around the left carina [8]. In this retrospective feasibility study, the aim was to report clinical outcomes and complications of the Y stents for bronchopleural fistula involving the upper carina.

## Methods

### Patients

From September 2011 to July 2017, 18 consecutive patients with fistulas were treated in our department for bronchial disease. The diagnosis of bronchopleural fistula was based on bronchoscopy and computed tomography (CT). The patients’ medical imaging and clinical records were reviewed. The main indication for this stenting technique was a bronchopleural fistula around the upper carina. All patients signed a written informed consent before the procedure. This retrospective study was approved by the institutional review board of Zhengzhou University First Affiliated Hospital.

### Preprocedure preparation

Routine clinical and laboratory examinations were performed before the procedure. A 64-slice unenhanced CT (SOMATOM Force CT, SIEMENS, Germany) was used for chest CT with scanning points from neck to thorax. The raw data was reconstructed by ADMIRE (strength = 3) with a slice thickness of 1 mm. The DICOM data were sent to a singo.via (VB10b) workstation (Siemens Heathineers). The diameter and length of the trachea and bronchi were measured in the singo.via workstation by two doctors with more than 10 years’ experience in airway disease. All of the Y stents were designed and manufactured according to individual airway dimensions (Micro-Tech Co. Ltd., Nanjing, China). Stents were woven with a nickel–titanium wire, with a diameter 10–20% larger than the airway diameter. The main body of the stent and its bronchial limbs were covered with polyethylene membranes. Antibiotic treatment was given for 3 to 7 days to control lung infection according to the results of bacterial culture and sensitivity testing.

### Stenting procedure

**Fig. 1:**
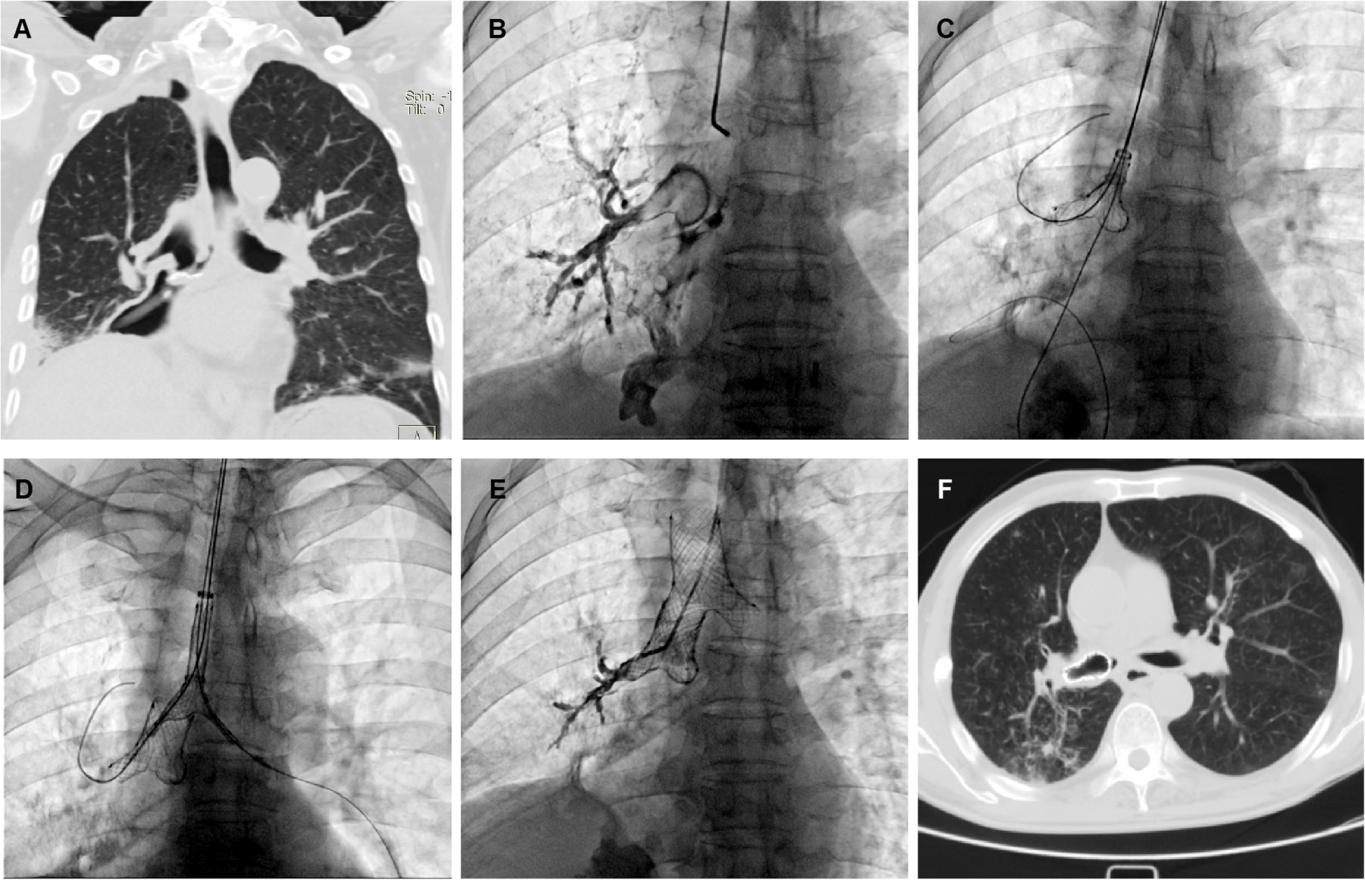
Stenting procedure for fistula in right middle lobe bronchus. **a** Two stiff guide wires were inserted into the distal end of the lobar bronchus and fistula. **b** A delivery system of small Y stent was introduced into the corresponding lobar bronchi. **c**, The branches of the small Y stent was deployed by retracting the two binding threads, and the main body of the stent was deployed by withdrawing the covering sheath. **d** The small Y stent was inserted to seal the airway fistula. Arrow indicates the fistula. **e** Radiography was performed again to confirm the seal of fistula after stent placement. **f** Chest SCT showed the sealing of bronchopleural fistula

**Fig. 2:**
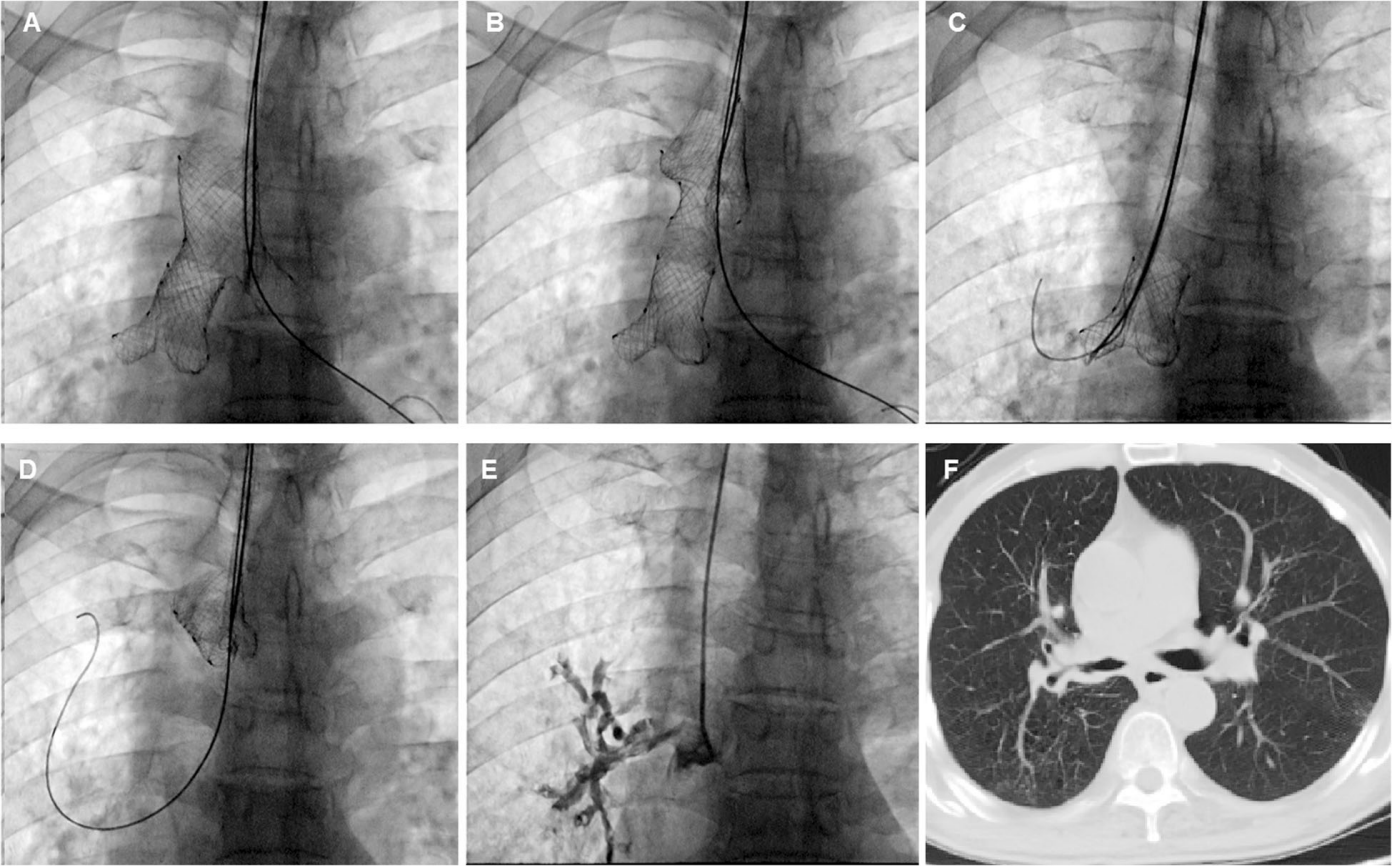
Stent removal after successful treatment. **a** The tip of the hook was placed next to the end of the large Y stent. **b** Metal Y stent was withdrawn from the airway wall. **c** The tip of the hook was placed next to the end of the small Y stent. **d** Small Y stent was withdrawn from the airway wall. **e** Radiography was performed again to confirm the seal of fistula immediately after stent removal. **f** Chest SCT showed the sealing of bronchopleural fistula and the disappearance of residual chest cavity

All of the airway stents were implanted under fluoroscopy rather than rigid bronchoscopy similar as previous report [9]. The patients were supine on the examination table, with the monitor of electrocardiogram. A 5F vertebral artery catheter (Cook Corporation, Bloomington, USA) was introduced into the diseased bronchus. The size and location of the fistula was shown after injection of contrast agent. The catheter was introduced into the distal end of the lobar bronchi of the diseased airway, and two stiff guide wires were inserted into the distal end of the lobar bronchi (Fig. 1, A). A delivery system of small Y stent was introduced into the corresponding lobar bronchi through a guide wire (Fig. 1, b). The branches of the small Y stent were deployed by retracting the two binding threads, and the main body of the stent was deployed by withdrawing the covering sheath (Fig. 1c, d). Similarly, a large Y stent was implanted into the main carina to overlap with the main body of the small Y stent. For the removal of airway stent, a 10F long sheath and retrieval hook was inserted (Fig. 2a, c). The tip of the retrieval hook was placed next to the end of airway stent to hook the metal wire firmly and withdraw the stent from the airway wall (Fig. 2b, d). Radiography was performed after stent placement to confirm the sealing of fistula (Fig. 2e; Fig. 4b).

### Follow up and definitions

Chest CT and/or airway radiography were used to confirm sealing of the bronchopleural fistula and stent patency during follow up (Fig. 1f; Fig. 2f; Fig. 4c). Flexible bronchoscopy was routinely performed and treatment was administered when restenosis or mucus obstruction is found. (Fig. 4**d**, **e**). Reintervention procedure such as stent removal was conducted once a severe complication was observed. Major complication is defined as perioperative death, stent migration, stent intolerance, restenosis and other causes leading to stenting failure or stent removal. Minor complications are defined as complications other than major complications, such as mucus retention and mild restenosis without stent removal. Stent restenosis is defined as reduction of stent lumen area due to tumor ingrowth or excessive granulation tissue, which results in a high risk of removal due to embedding of the stent.

## Results

### Patient demographics

All patients underwent lobectomy due to lung cancer (*n* = 14) or benign lung disease (*n* = 4). Eight patients underwent middle-lower bilobectomies and six patients underwent right middle lobectomy. One patient each underwent right upper lobectomy, left upper lobectomy, right lower lobectomy and left lower lobectomy, respectively. After a median interval of 0.5 month (Interquartile range, IQR 0.3–1.5), patients showed airway symptoms for a median symptom duration of 0.8 month (IQR 0.4–2.6). Sixteen patients developed a bronchopleural fistula around the right upper carina and two patients showed a fistula around the left upper carina (Table 1). Twelve patients presented with symptoms of cough and/or vomiting and 3 of these patients also were febrile. In addition, three patients were only febrile and three patients had no obvious symptoms, but drainage of purulent fluid was observed.

**Table 1:** Patient demographics

Demographics	Median (IQR) or No.
Patients, No.	18
Age, years	61.0 (50.8–65.8)
Male gender	18
Duration of symptom, Months	0.8 (0.4–2.6)
Interval between surgery and symptom, Months	0.5 (0.3–1.5)
Previous disease	
Lung cancer	14
Benign lung disease	4
Location of fistula	
Right up lobe bronchus	1
Right middle lobe bronchus	15
Left bronchus	2

### Stenting procedure

**Fig. 3:**
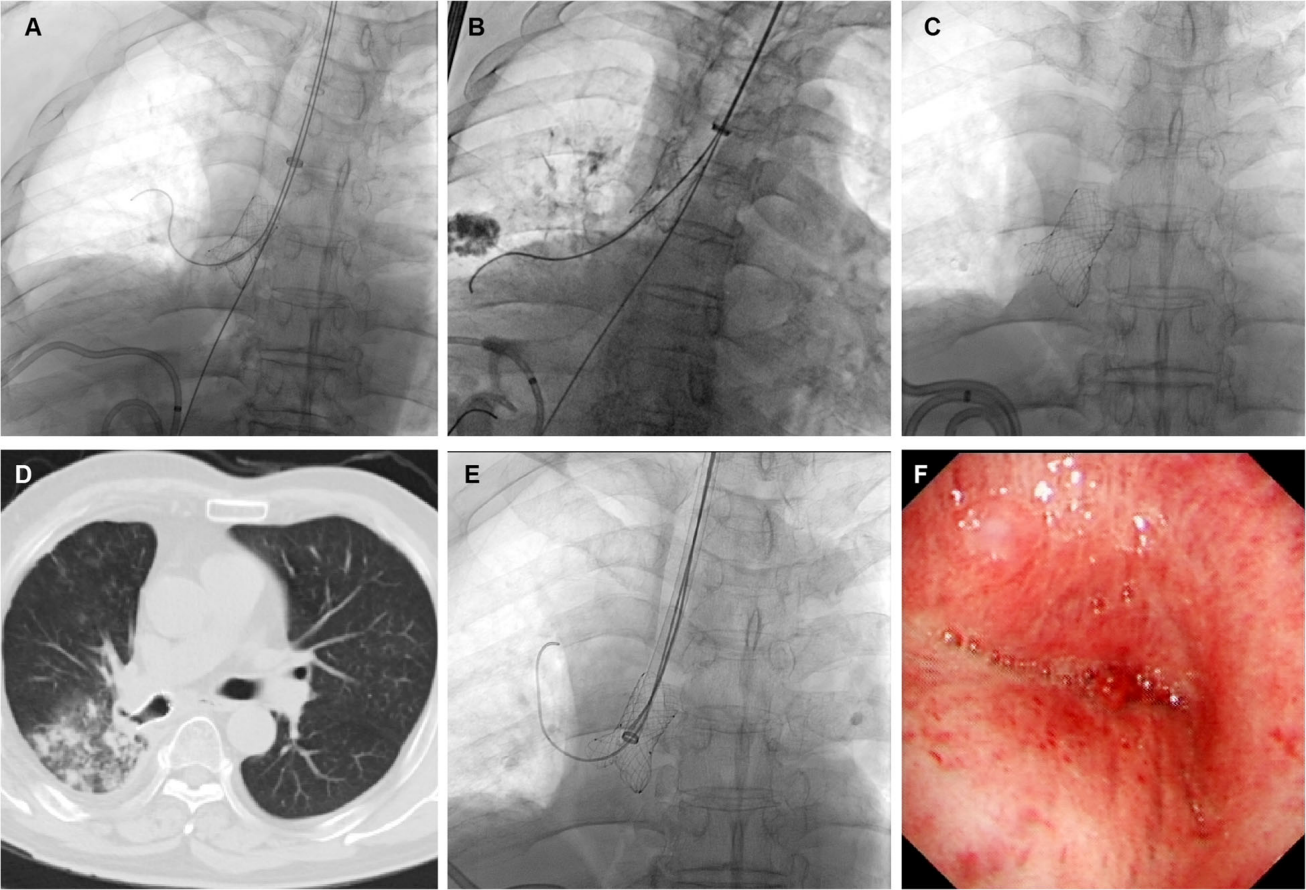
Treatment of a case with fistula in right middle lobe bronchus. **a** A bronchopleural fistula was treated by small Y single-plugged airway stent. **b** The stent was migrated during withdrawing the guide wires. **c**, **d** After the second procedure, the stent was implanted successfully and the fistula healed. **e** The Y stent was withdrawn after 105 days. **f** Bronchoscopy confirmed the sealing of bronchopleural fistula

The stents were implanted successfully for the first time in 17 patients. One patient failed at the first attempt because the stent migrated during withdrawal of the guide wires (Fig. 3a, b). After the second procedure, the stent was placed and the fistula healed successfully (Fig. 3c-f). A total of 19 small Y single-plugged stents and 5 large Y stents were inserted (Table 2). Airway radiography performed immediately after the procedure revealed that the fistulas were fully sealed. Twelve patients underwent successful stent removal due to complications or cure, for median stent duration of 89.5 days (IQR 45, 104.8). These stents were removed successfully for the first attempt with no major complication.

**Table 2:** Types and measurement of individual airway stents

Types of individual airway stents	No.	Median diameter (IQR)	Median length (IQR)
Small Y single-plugged stents, mm	19	MB 17 (15.3, 19.5)	MB 17.5 (11.3, 21.5)
		BL 10 (10, 12)	BL 10 (10, 14.3)
		PL 12 (10, 14.8)	PL 10 (10, 14.3)
Large Y stents, mm	5	MB 22 (20, 24)	MB 30 (27.5, 32.5)
		RMB 14 (13, 17.5)	RMB 15 (12.5, 17.5)
		LMB 16 (15, 17)	LMB 10 (10, 18)

MB, main body; PL, Plugged bullet limbs; BL, bronchial limbs; LMB, left main bronchus; RMB, right main bronchus

### Complications and stent removal

**Fig. 4:**
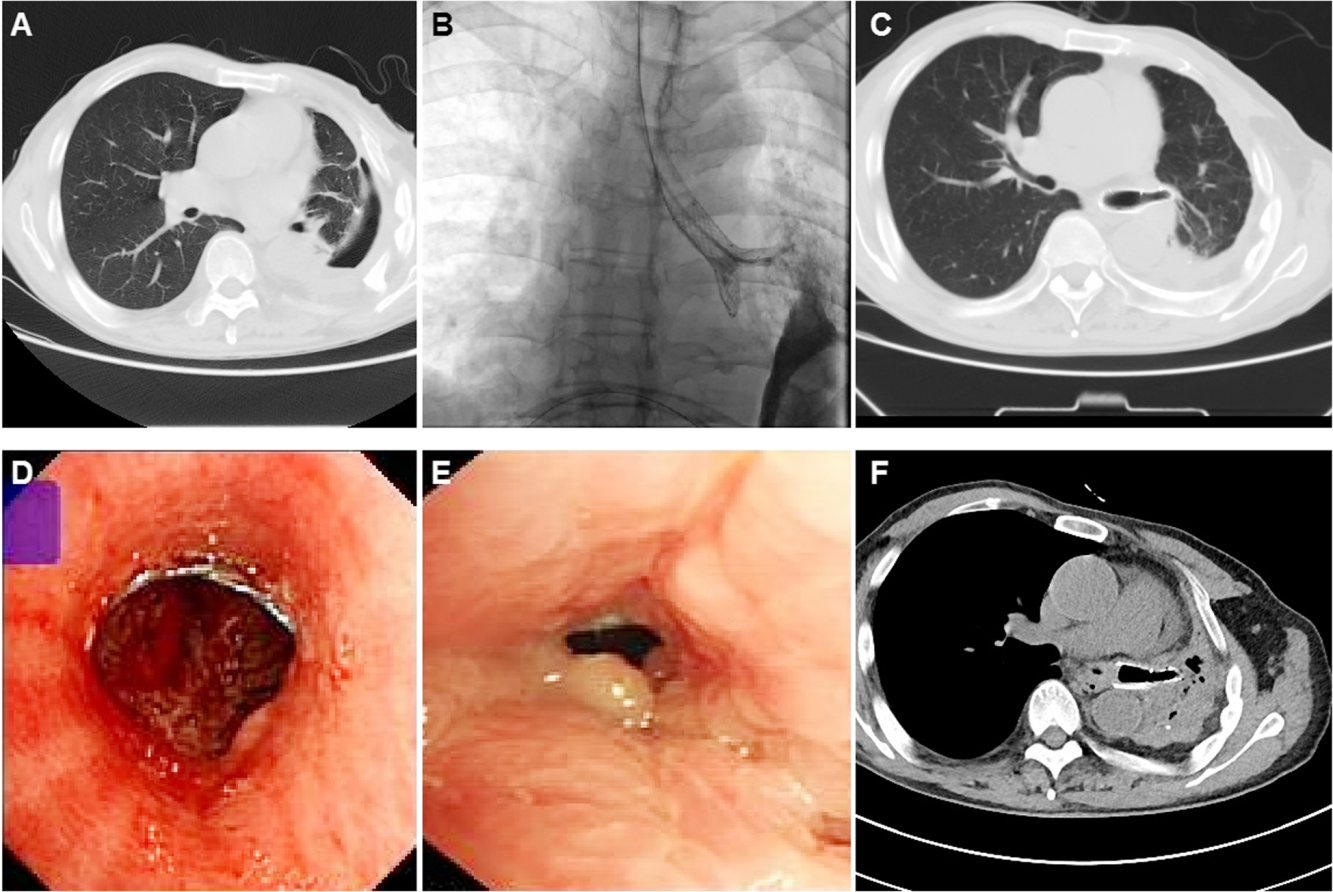
Treatment of a case with fistula in left low lobe bronchus. **a** A bronchopleural fistula was shown by chest SCT before stenting. **b** A small Y stent is inserted in the left upper carina. **c**, Chest SCT showed an obvious decreased residual chest cavity 1.5 months after stenting. **d** Bronchoscopy confirmed the patency of stent 1.5 months later. **e**, **f** Occluded stent and left atelectasis was found 34.4 months after stent placement owing to the stent was not removed in time

One case died of ventricular fibrillation the second day after the procedure. No other severe complications such as massive hemorrhage or airway rupture occurred during the procedure. Nineteen complications were observed in 14 patients; including 4 major complications (see Table 3). At 15 days after the procedure stent migration occurred in one patient and the migrated stent was removed. One patient complained of stent intolerance 4 days post-procedure and this stent was removed. The remaining 17 patients tolerated the stent well and had a good palliation of airway symptoms although these complications occurred. One patient showed an obvious decreased residual chest cavity 1.5 months after stent placement. An occluded stent and left atelectasis was found 34.4 months after stent placement owing to the stent was not removed in time (Fig. 4f). Since there were no symptoms, no further treatment was needed. Eight patients had pneumonia during stent placement and this was considered a minor complication as the result of mucus retention after stenting. Endoscopic saline lavage and mucus aspiration was performed for patients with mucus obstruction. Eight patients showed stent restenosis during follow up. Argon knife or cryotherapy, high-frequency electrosurgical excision was performed to help restore airway patency.

**Table 3:** Clinic effect and total complications of stenting

	N	Median days after stenting (IQR)
Clinic efficacy		
Cure	9	31.8 (25.6, 34.4)
Improved	3	82 (62.9, 122.8)
Death	5	9 (3.9, 53.4)
Total complications		
Death	1	1
Stent restenosis	8	97 (69, 111.5)
Intolerance of stenting	1	4
Stent migration	1	15
Retention of mucus	8	55 (19.5, 87)
Pneumonias	8	55 (19.5, 87)

### Follow up

As noted previously, one patient died due to ventricular fibrillation the second day after the procedure. Of the remaining 17 patients, 1 patient lost follow-up and 16 cases were followed up for a median time of 31.8 months (range 13.6–72.5 months). Nine patients were cured, the fistula was sealed, the residual chest cavity disappeared, and patients were asymptomatic. Three cases were clinical improved, with an obvious decreased residual chest cavity and remission or disappearance of symptoms. Nine patients presented with pneumonia before stent placement, and 8 patients showed pneumonia while the stent was in place. Except for the one perioperative death, four patients died of tumor 7.8 to 91.7 months during follow up after stenting. The 1-, 3-, and 5-year survival rates were 87.5, 80.8 and 80.8%, respectively.

## Discussion

Currently, few studies have reported the use of metallic Y stents for bronchopleural fistula around the upper carina. Our study demonstrated that metallic Y stent placement is feasible and provides immediate sealing of the fistula with good long-term efficacy. Stent placement was successful for the first time in 17 patients. Nineteen complications were observed in 14 patients, and were considered major complications in 4 patients. No other severe complications such as massive hemorrhage or airway rupture occurred during the procedure.

Different types of airway stents have been studied, including Y silicone airway stents [9–11] and self-expanding metallic airway stents [12–17]. The silicone airway stent is one of the most common types of stent, and often shows advantages of easy removal, durability and low cost when compared with metallic stents [7, 9, 18–20]. Silicone Y-stents have been inserted in the main carina and the right upper carina [7, 11, 21–23], and the secondary left carina [8, 10]. Metallic Y stents, on the other hand, show good support and flexibility, can adhere to the bronchus, and can be inserted with the help of guide wire. Metallic stent placement may be less traumatizing and minimize the procedures in cases with a fistula [8]. Currently, metallic Y stents are available and used clinically. Individualized metallic Y stents can be produced upon request and are designed to fit on the main carina [9]. Besides, silicone stent can be placed under a rigid bronchoscope by a respiratory physician. Metallic airway stent can be placed under rigid bronchoscopy or under fluoroscopy.

In the present study, customized small metallic Y stents were used for fistulas around the upper carina. The main indication for placement of a metallic Y-shaped covered stent was a bronchopleural fistula around the upper carina. For this kind of fistula, a traditional tubular stent or large Y stent may not be able to effectively plug the fistula. A small Y single-plugged stent is inserted in the upper carina to plug the fistula, and large Y stents also may be used in the main carina to overlap with the small Y stent to further improve the plugging effect and prevent stent migration. Based on our experience, metallic Y stents may well be an alternative to silicone Y stents for lesions around the upper carina. In addition, implanted Y stents show excellent stability and a low migration rate [24]. Owing to its anti-migration properties, use of the Y stent in the upper carina may be an alternative to the conventional straight stent.

The limitation is that this is a retrospective study with a small sample size. A larger prospective study is necessary to further study the outcome of this procedure. In addition, our department has extensive experience in metallic Y stent placement and removal. These good results of this study might not be reproduced by less experienced personnel. However, if a Y stent with an appropriate diameter and length is selected, the placement does not seem to be too difficult.

## Conclusions

Metallic Y stenting is technically feasible, effective and safe for bronchopleural fistula around the upper carina.

## Data Availability

The datasets used and/or analyzed during the current study are available from the corresponding author on reasonable request.

## Abbreviations

CT: Computed tomography
IQR: Interquartile range
